# A Rare Case of Resistant Hyperkalemia: Unveiling Biktarvy’s Impact on Renal Function in HIV Management

**DOI:** 10.51894/001c.144535

**Published:** 2025-09-30

**Authors:** Srishti Kanda

**Affiliations:** 1 Department of Internal Medicine Detroit Medical Center-Sinai Grace

## 91

### INTRODUCTION

Resistant hyperkalemia presents a significant challenge in patients with multiple comorbidities, particularly those on antiretroviral therapy. Hyperkalemia, characterized by elevated serum potassium levels, can be life-threatening and is often associated with renal dysfunction or medication use. Antiretroviral therapy, essential for HIV management, can sometimes contribute to hyperkalemia. This case report describes a rare instance of refractory hyperkalemia induced by Biktarvy in a patient with HIV.

### CASE PRESENTATION

A 56-year-old female with HIV, managed with Biktarvy since 2004, was admitted to the emergency department on May 23, 2024, with acute shortness of breath and was diagnosed with pneumonia. During hospitalization, she developed acute kidney injury (AKI) on chronic kidney disease (CKD) stage IIIb and resistant hyperkalemia. Her baseline glomerular filtration rate (GFR) was 44, and serum creatinine peaked at 2.3 mg/dL from an initial 1.4 mg/dL. Nephrology was consulted due to concerns about nephrotic syndrome, potentially secondary to focal segmental glomerulosclerosis (FSGS) related to her HIV status. However, FSGS was ruled out due to the absence of nephrotic-range proteinuria.

### DISCUSSION

Management included dietary potassium restriction, sodium polystyrene sulfonate, and sodium zirconium cyclosilicate, along with close monitoring. Given concerns that Biktarvy was exacerbating hyperkalemia, the medication was discontinued. A thorough workup, including plasma renin and aldosterone levels, was conducted. The patient’s potassium levels gradually normalized to 4.3 mEq/L, and she was discharged with a renal-friendly diet and nephrology follow-up. At discharge, her renal function showed improvement, and her HIV regimen was adjusted to individual components—Tenofovir TAF, Emtricitabine, and Dolutegravir—rather than the combination pill.

### CONCLUSION

This case highlights hyperkalemia as a rare but clinically significant side effect of Biktarvy, particularly in patients with underlying renal impairment. Bictegravir, a key component of Biktarvy, is believed to disrupt renal potassium handling, increasing hyperkalemia risk in susceptible individuals. The resolution of hyperkalemia upon discontinuation of Biktarvy supports its role in exacerbating the condition. This case underscores the importance of early recognition, multidisciplinary management, and individualized treatment plans to balance renal function and effective HIV therapy in complex patient populations.

